# Molecular and metabolic pathways mediating curative treatment of a non-Hodgkin B cell lymphoma by Sindbis viral vectors and anti-4-1BB monoclonal antibody

**DOI:** 10.1186/s40425-019-0664-3

**Published:** 2019-07-15

**Authors:** Minjun Yu, Iris Scherwitzl, Silvana Opp, Aristotelis Tsirigos, Daniel Meruelo

**Affiliations:** Perlmutter Cancer Center at NYU Langone Health, NYU Gene Therapy Center, and Department of Pathology, NYU School of Medicine, 550 First Avenue, New York, NY 10016 USA

**Keywords:** Sindbis virus(SV), α4-1BB, T cell, IFNγ, RNA-Seq, IVIS imaging

## Abstract

**Background:**

Limitations to current therapies for treating non-Hodgkin B cell lymphoma include relapse, toxicity and high cost. Thus, there remains a need for novel therapies. Oncolytic viral (OV) therapy has become a promising cancer immunotherapy because of its potential effectiveness, specificity and long-lasting immunity. We describe and characterize a novel cancer immunotherapy combining Sindbis virus (SV) vectors and the agonistic monoclonal antibody (mAb) to the T cell costimulatory receptor, 4-1BB (CD137).

**Methods:**

A20 lymphoma was transfected with luciferase and tumor cells were inoculated to BALB/c mice. Tumor growth was monitored by IVIS imaging. Tumor bearing mice were treated with Sindbis virus, α4-1BB Ab or SV plus α4-1BB Ab. On day 7 after treatment, splenocytes were harvested and surface markers, cytokines, and transcription factors were measured by flow cytometry or Elispot. Splenic T cells were isolated and RNA transcriptome analysis was performed. Tumor cured mice were rechallenged with tumor for testing immunological memory.

**Results:**

SV vectors in combination with α4-1BB monoclonal antibody (mAb) completely eradicated a B-cell lymphoma in a preclinical mouse model, a result that could not be achieved with either treatment alone. Tumor elimination involves a synergistic effect of the combination that significantly boosts T cell cytotoxicity, IFNγ production, T cell proliferation, migration, and glycolysis. In addition, all mice that survived after treatment developed long lasting antitumor immunity, as shown by the rejection of A20 tumor rechallenge. We identified the molecular pathways, including upregulated cytokines, chemokines and metabolic pathways in T cells that are triggered by the combined therapy and help to achieve a highly effective anti-tumor response.

**Conclusions:**

Our study provides a novel, alternative method for B cell lymphoma treatment and describes a rationale to help translate SV vectors plus agonistic mAb into clinical applications.

**Electronic supplementary material:**

The online version of this article (10.1186/s40425-019-0664-3) contains supplementary material, which is available to authorized users.

## Background

Chemotherapy and immunotherapy (monoclonal antibodies (mAbs) and CAR-T therapy) have been used to treat non-Hodgkin B cell lymphoma. For both conventional chemotherapy and immunotherapy, tumor relapse is a common problem [1]. Establishment of a potent, safe, but also long-lasting immune response is a major goal of B cell lymphoma treatment. CAR-T therapy is a novel immune therapy used to treat diffuse large B cell lymphoma. However, major drawbacks to current CAR-T therapy include: risk of immune incompatibility for allogeneic CAR-T [2]; quality control for harvesting self T cells; time consumption for processing to autologous CAR-T [3]; off-target effects; the possibility that the treatments causes a cytokine storm and high cost. Therefore, there is a need to investigate alternative and reliable methods for treating B cell lymphoma.

Oncolytic virus (OV) therapy has become a novel immunotherapeutic approach to treat cancer. A rationale for oncolytic virus is that they can infect and lyse the tumor cells [4]. They have been made to selectively replicate in tumor cells either through the direction of tumor specific promoters or through direct intratumoral administration. Most OVs encounter a number of barriers to systemic administration. Once lysed by OVs, tumor cells release tumor associated antigens (TAAs) [5] that can stimulate cytotoxic T cells. OV infection also induces an inflammatory response that helps to trigger an immune anti-tumor response [6]. Several OV clinical trials are underway and have shown promising results [7]. However, whether OV therapy can effectively treat tumors that they are unable to infect remains an unresolved limitation.

Sindbis virus (SV) belongs to alphavirus genus and is one type of OV [4, 8]. Though it does not lyse infected tumor cells, it can cause their apoptotic death. It offers several important benefits. SV is known as one of the least virulent alphaviruses with clinical signs and symptoms usually unapparent [9]. It has been estimated that there are 17 times more subclinical than symptomatic SV infections [10]. In general, when symptoms do occur in humans they consist of a self-limiting, mild, febrile disease with vesicular exanthema and arthralgia from which most patients recover within 14 days [11]. The disease is in part self-limiting because SV is an RNA virus that does not integrate in the host genome and hence its presence is transitory [12]. The lack of an integrative step in its replication cycle also avoids insertional mutagenesis risks. In addition, our SV vectors were generated from the laboratory strain AR339, which is not known to cause disease in humans [13]. We further attenuated these vectors by rendering them replication-defective [14].

SV vectors can target tumors systemically and can reach metastatic tumor cells throughout the body. They can target tumors without infecting normal tissues [8]. However, susceptibility to infection by SV vectors depends on a number of factors including laminin receptor expression [15] and distribution, as well as, defects in IFN signaling in tumors [16]. Here we document that SV vectors can effectively help cure tumors that they are unable to infect.

Our present studies use an antibody directed at 4-1BB (CD137, TNFRSF9), a T cell costimulatory molecule. 4-1BB agonist stimulation greatly enhances NK and cytotoxic T cell activity. There are preclinical studies showing that α4-1BB effectively treats lymphoma and that depletion of Treg cells enhances the therapeutic effect of α4-1BB [17]. The A20 tumor cells we use in the present study were derived from a spontaneously arising reticulum cell sarcoma (a non-Hodgkin lymphoma) in a BALB/c mouse.

Previously, we used SV carrying NYESO-1, which encodes the cancer testis TAA, NYESO-1, to cure CT26 tumors expressing NYESO-1 [18]. Here we show that systemically disseminated A20 lymphoma can be completely cured by SV plus α4-1BB mAb combination therapy without the need to produce a SV that encodes a TAA known to be present in the A20 lymphoma cells. Further, neither intratumoral injection of the SV vectors nor infection of the tumors is required as the A20 B lymphoma cells used in the current model are resistant to SV infection.

One difference in the current study, compared with those we previously published, is the use of SV vector combination therapy that involves an agonistic mAb for a costimulatory receptor versus targeting checkpoint blockade molecules such as CTLA4 and PD-1. Here we show that agonistic mAbs in combination with SV vectors trigger a cascade of events that results in curative results.

Our findings reveal the potential of SV combination therapy to cure tumors for which TAAs are completely unknown.

## Methods

### Firefly luciferase (Fluc)-expressing A20 cells generation

A20 cells were transfected with pGL4-neo_Fluc plasmid (Promega) by electroporation via Nucleofector™ kit V (Lonza). Fluc-A20 cell clones were selected and maintained in RPMI1640 (Cellgro) + 10% FBS (Gibco) + 250 μg/ml G418 (Gibco). One A20 clone stably expressed fLuc and was used for tumor inoculation and consecutive experiments.

### SV production

SV-LacZ production and titering were done the same as previously described [18].

### SV-GFP infection

A20 cells and control BHK cells were infected by SV carrying GFP for 1 h. The GFP expression was observed the next day by fluorescence microscopy.

### A20 tumor inoculation and In Vivo Imaging System (IVIS) imaging

3 × 10^6^ fLuc-A20 cells were inoculated to BALB/C mice by i.p injection. Tumor growth was monitored as previously described [18].

### SV and α4-1BB Ab treatment

Treatment was started after successful tumor inoculation (4 days after tumor cell injection, confirmed by IVIS imaging). Tumor growth was measured every week by noninvasive bioluminescent imaging. SV-LacZ was injected 4 times per week, for totally 3 weeks. The virus (10^7^–10^8^ TU/mL) in a total volume of 500 μL was i.p. injected. For 2 groups (41BB and SV plus 41BB), 350 μg/mouse 41BB Ab was injected 3 times/week for 2 weeks. InVivoMAb anti-mouse 4-1BB was ordered from BioXCell (Clone: LOB12.3, Cat.No. BE0169). In low dose treatment protocol, SV-LacZ was injected i.p. 3 times per week, for totally 3 weeks. 41BB Ab (50 μg/mouse) was injected once a week for 3 weeks.

### Elispot

Mouse IFNγ ELISPOT was performed according to the manufacturer’s protocol (BD Biosciences). 2 × 10^5^ splenocytes or 1 × 10^5^ T cells were plated per well O/N in RPMI supplemented with 10% FBS. For a positive control, splenocytes were stimulated with 5 ng/ml PMA + 1 μg/ml Ionomycin.

### Flow cytometry

Fluorochrome-conjugated antibodies against mouse CD3, CD4, CD8, CD25, CD44, CD62L, ICOS, CD11a, ICAM-1 were purchased from Biolegend (San Diego, CA). Fluorochrome-conjugated antibodies against mouse Foxp3, EOMES and CCR5 were purchased from Thermofisher. BUV395 conjugated antibody against mouse CD8a was purchased from BD Biosciences. For surface staining, cells were washed and stained with anti-mouse direct conjugated antibodies. Cells were analyzed using the LSRII flow cytometer (BD Biosciences) and data were analyzed using Flowjo software (Treestar, Ashland, OR). For intracellular cytokines staining, stimulated cells were fixed with cytofix/cytoperm solution (BD Biosciences), permeablized with perm/wash buffer (BD Biosciences) and stained with anti-mouse IFNγ antibodies. For nuclear antigen, cells were fixed and permeabilized by Foxp3 fixation/permeabilization buffer (eBioscience) and stained with anti-Foxp3, T-bet, Ki67 and EOMES antibody.

### RNA isolation and transcriptome analysis

Total RNA was harvested by RNAeasy isolation kit (Qiagen, Valencia, CA). For each group, 3 BALB/C mice were used as biological repeats. RNA-seq was performed by NYUMC Genome Technology Center (GTC). To identify significant differences in expression between any pair of groups, differential expression analysis was performed using Deseq2 and an adjusted *p* value cutoff of 0.05 was applied [19] (q < 0.05). To increase stringency, only genes with a Log2 fold change≥1 (upregulated) or ≤ − 1 (downregulated) were selected for further analysis. Gene cluster analysis was performed by DAVID analysis using the selected differentially expressed genes [20, 21]. RNA-seq results (normalized counts) were used as input to perform with Gene Set Enrichment Analysis (GSEA) [22]. Molecular Signatures Database (MSigDB)v4.0 were used as screening database. For each gene, the gene expression value is normalized by the relative log2 fold change compared to the median value of this gene. Expression heatmap is drawn by Morpheus (https://software.broadinstitute.org/morpheus/). Cannonical pathway and disease and biological functional analysis were generated by ingenuity pathway analysis (IPA; Ingenuity Systems, Redwood City, CA) using the statistical differential expressed genes list. To increase the sample representativeness, for IPA, we choose nominal *p* < 0.05 as cutoff value.

### Tumor infirtrating lymphocyte (TIL) harvest

To investigate the phenotype of TIL, all treatments were started 11 days after tumor inoculation, After 7 days treatment, tumor mass was harvested and the phenotype of TIL were analyzed as previously described [18].

### T cell seahorse assay

T cells were isolated from spleen by using pan T cell isolation kit (Stemcells). T cells were plated at 6 × 10^5^ cells/well in 24 well plate. Oxygen consumption rate (OCR) and excellular acidification rate (ECAR) were measured by Agilent Seahorse XFe24.

### Statistical analysis

For the two group comparison, statistical difference was determined by unpaired two tail Student t-test. The multiple sample comparison was analyzed by one way ANOVA. *P* < 0.05 was determined to be significant for all experiments. All values were calculated with Excel (Microsoft) and Prism software (GraphPad).

## Results

### SV and α4-1BB mAb combination completely cured A20 lymphoma

To explore if SV has therapeutic effect on tumors not targeted or infected by SV vectors, we used the A20 B cell lymphoma, which is highly resistant to SV infection (Additional file 1: Figure S1).

**Fig. 1:**
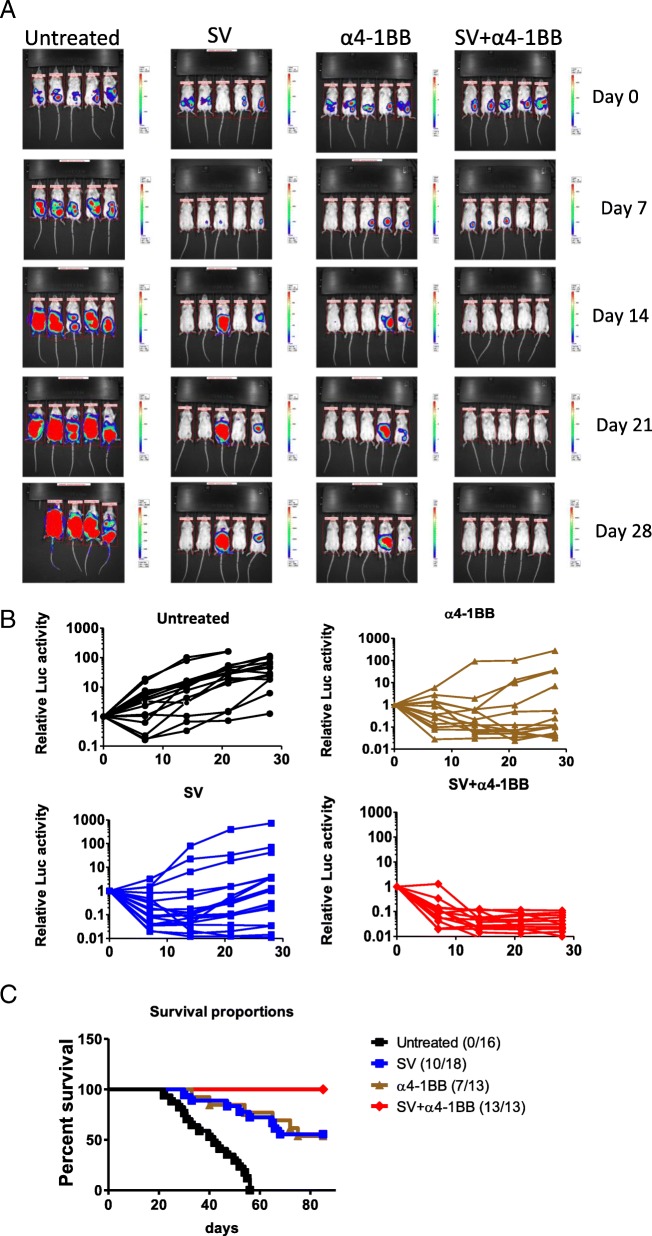
Sindbis virus (SV) and α4-1BB combination completely cured BALB/C mice A20 lymphoma. **a** Representative bioluminescence images of groups as indicated. Intensity scale, day 0, 7, 21, min: 400, max:7000; day 14, min: 100, max: 1000; day 28, min: 3000, max: 50000. **b** Tumor growth was measured by relative firefly luciferase (fLuc) activity (normalized to day 0 fLuc activity). Untreated, *n* = 16; SV, *n* = 18; α4-1BB Ab, *n* = 13; SV plus α4-1BB Ab, *n* = 13. **c** Survival curve of all groups (the ratio is shown as survived number/total number)

To monitor tumor growth in vivo, a firefly luciferase (f-Luc) expression vector was transfected into the A20 lymphoma cell line by electroporation. A stable f-Luc expressing A20 clone was isolated through G418 selection. We inoculated 3 × 10^6^/mouse f-Luc A20 tumor cells by intraperitoneal (i.p.) injection. Tumor growth was monitored by IVIS imaging once per week. Tumors were successfully established after 4 days inoculation (Fig. 1a). After tumors were established, SV and α4-1BB mAb treatment started (designated as day 0). We used a therapeutic protocol similar to that previously described [18]. SV plus α4-1BB mAb combination achieved the best therapeutic effect (Fig. 1b). All mice in that group showed complete tumor regression in 2 weeks. Although both SV or α4-1BB treatments alone achieved obvious therapeutic effects compared with untreated mice, they were not as effective as the combination and a fraction of mice in these two groups eventually succumbed to tumor (Fig. 1c).

### SV alone and SV plus α4-1BB mAb stimulated cell cycle progression, cytokine production, and activation

**Fig. 2:**
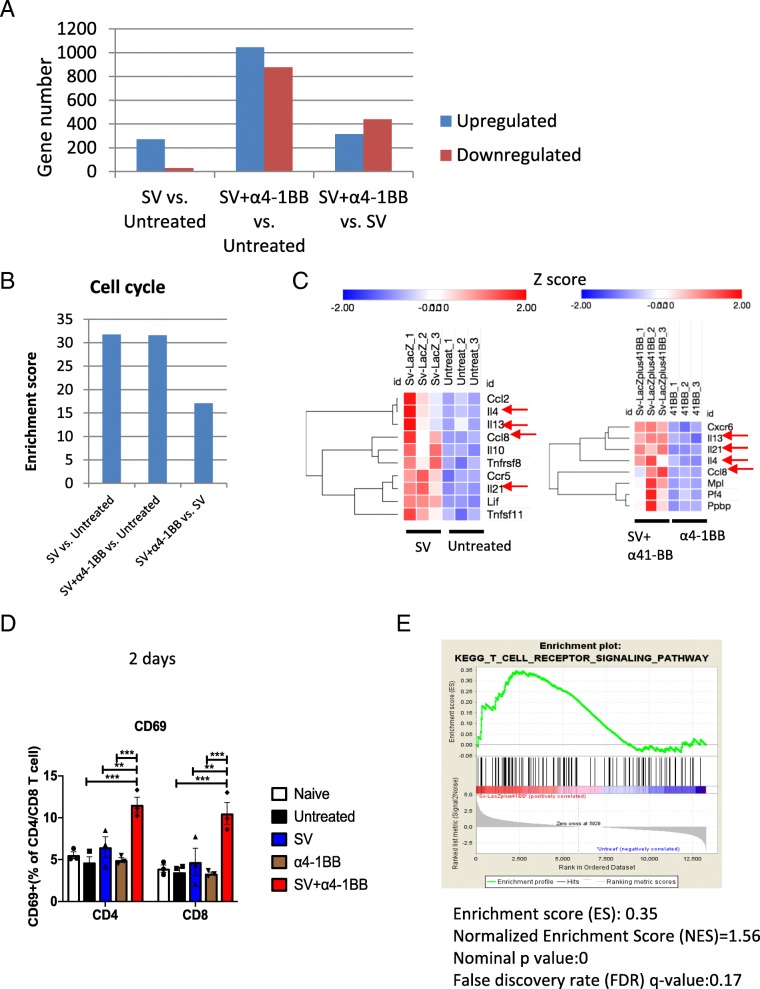
SV alone and SV plus α4-1BB mAb stimulated cell cycle progression, cytokine production, and activation. **a** The numbers of significant differential (SD) expressed genes (upregulated and downregulated) of SV vs. untreated are as indicated. SD expressed genes were selected based on Deseq2 analysis (q < 0.05), |Log_2_FC| ≥ 1. **b** The enrichment scores for gene cluster of cell cycle for SV vs. untreated, SV+ α4-1BB vs. untreated and SV+ α4-1BB vs. SV respectively (“cell cycle” is the gene cluster with the highest enrichment score for these 3 comparisons). **c** The heat map representing SD expressed cytokine and chemokine genes (left, SV vs. untreated; right, SV+ α4-1BB vs.α4-1BB, Log2FC ≥ 1). Expression values are shown by Z-score. Genes are hierarchically clustered by one minus Pearson correlation. Red arrow, Ccl8, IL4, IL13 and IL21 expression. **d** The percentage of CD69^+^ T cells from all groups on day 2 after starting treatment was measured by flow cytometry. **e** GSEA enrichment plot of KEGG (SV + α4-1BB vs. untreated) TCR receptor signaling pathway. *, *p* < 0.05; **, *p* < 0.01, ***, *p* < 0.001

In our study, SV significantly inhibited tumor growth by day 7 (Fig. 1a). T cells play a critical role in SV induced anti-tumor immunity. T cell response reaches a peaked on day 7 after infection [18]. To explore how SV induced T cell responses that help to eradicate A20 lymphoma, RNA-Seq was performed using purified splenic T cells from all groups on day 7. Compared with untreated samples, we identified 271 genes upregulated (q < 0.05 and Log2 Fold Change≥1) and 28 genes downregulated (q < 0.05 and Log2 Fold Change≤ − 1) in the SV infected group through Deseq2 analysis (Fig. 2a, Additional file 2: Table S1). NIH DAVID cluster analysis was performed using the upregulated gene list. Enriched clusters were ranked based on enrichment score. Cell cycle gene cluster achieved the highest enrichment score (Fig. 2b, Additional file 1: Figure S2A). This result was confirmed by KEGG gene set enrichment analysis (GSEA) (Additional file 1: Figure S2B). Cell cycle gene set ranks as the highest (enrichment score = 0.64, FDR q value = 0.1, nominal *p* value = 0). These results indicate that SV infection enhances T cell cell cycle progression. SV induced upregulation of a series of cytokine and chemokine/chemokine receptors (Fig. 2c, left). To identify cytokines/chemokines that are upregulated by the administration of SV vectors, we compared SV plus α4-1BB mAb versus α4-1BB mAb (Fig. 2c, right). CCL8, IL-4, IL-13 and IL-21 were among those RNAs whose expression was upregulated by SV treatment. IL-21 anti-tumor effect is dependent on the activation of T, B and NK cells [23]. IL-4, IL-10, IL-21 upregulation is consistent with previous reports [24, 25].

In addition, Ingenuity Pathway Analysis (IPA) indicates that SV treatment enhances T cell movement by altering the expression of a number of molecules involved migration (Additional file 3: Table S2, Additional file 1: Figure S2C), including a number of chemokines and chemokine receptors.

To understand why SV plus α4-1BB mAb achieves the best therapeutic effect, we ran Deseq2 analysis for SV plus α4-1BB mAb vs. untreated samples. We identified 1046 upregulated genes (q < 0.05 and Log2 Fold Change≥1) and 877 downregulated genes (q < 0.05 and Log2 Fold Change≤ − 1) in the SV plus α4-1BB mAb group (Fig. 2a, Additional file 4: Table S3). We also compared T cells from animals treated with SV + α4-1BB mAb vs. treated with SV only and found 316 upregulated genes (*p* < 0.05 and Log2 Fold Change≥1) and 439 downregulated genes (*p* < 0.05 and Log2 Fold Change≤ − 1) in the SV + α4-1BB mAb treated group (Fig. 2a, Additional file 5: Table S4). Next, we ran NIH DAVID analysis using the upregulated gene list. In both comparisions, cell cycle genes upregulation is the highest enrichment cluster [although SV + α4-1BB mAb vs. SV has a lower enrichment score compared with SV plus α4-1BB mAb vs. untreated samples (Fig. 2b and Additional file 1: Figure S3). This indicates that SV + α4-1BB mAb induced more potent T cell cycle progression compared with SV only. T cell proliferation is critical for an effective anti-tumor response against A20 lymphoma. The CD4/CD8 T cell ratio in untreated mice decreased markedly by day 28 after tumor inoculation (Additional file 1: Figure S4A-B). In addition, Treg/CD8 T cell ratio increased by day 28, indicating impairment of T cell function (Additional file 1: Figure S4C-D). In other groups the T cell ratio remained constant due to proliferation.

CD69 is the earliest marker of immune system activation. SV plus α4-1BB mAb treatment synergistically upregulated CD69 on day 2 (Fig. 2d). Additionally, KEGG GSEA indicates that T cell receptor signaling gene sets were enriched when comparing SV + α4-1BB vs untreated samples (enrichment score = 0.35, Normalized Enrichment Score (NES) = 1.56, FDR q value = 0.17, nominal *p* value = 0) (Fig. 2e).

### SV plus α4-1BB mAb stimulated cytotoxic T cell function

**Fig. 3:**
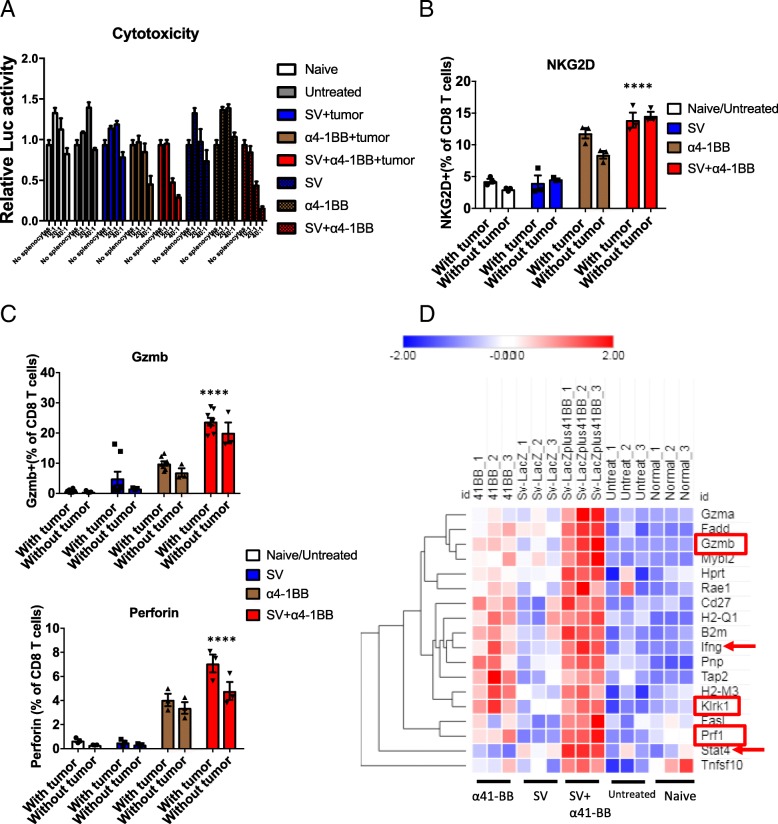
Sindbis virus plus α4-1BB combination induced higher cytotoxicity. **a** Splenocytes were mixed with fLuc-A20 lymphoma cells according to the ratio as indicated (splenocytes:lymphoma cells). Cytotoxicity corresponds to the reduction of normalized Luc activity (fLuc activity of A20 lymphoma cells only is normalized to 1). SV + tumor, α4-1BB + tumor, SV+ α4-1BB + tumor: tumor inoculated mice. SV, α4-1BB, SV+ α4-1BB: mice without tumor inoculation. **b** Splenocytes were harvested from all groups after 7 days treatment. The percentage of NKG2D^+^ cells was measured by flow cytometry (CD8 T cell gated). **c** The percentage of granzyme B+ and perforin+ cells was measured by flow cytometry (CD8 T cell gated). **d** Cytotoxicity associated genes were upregulated in SV + α4-1BB treated group. The heat map depicts the relative expression level of cytotoxicity associated genes. Expression values are shown by Z-score. Genes are hierarchically clustered by one minus Pearson correlation (day 7). Red square, granzyme b and perforin expression. Red arrow, Ifng and Stat4 expression. **, *p* < 0.01; ****,*p* < 0.0001

To investigate the antitumor cytotoxicity of SV/α4-1BB treated splenocytes, we co-cultured f-Luc A20 lymphoma cells with splenocytes on day 7. The ratios explored between splenocytes and tumor cell were 40:1, 20:1, 10:1. SV plus α4-1BB treated splenocytes demonstrated the highest cytotoxicity among all groups, as calculated by the reduction of f-Luc activity (Fig. 3a). To understand if this response is induced by TAA or anti-viral immunity, the same experiment was performed using mice under treatment but without tumor inoculation. We found that SV plus α4-1BB achieves the same effect as the combination treatment with tumor inoculation. This indicates that anti-tumor response on day 7 was not tumor specific. Accordingly, NKG2D, granzyme B and perforin were highly expressed in CD8 T cells from α4-1BB treated mice. In addition, SV plus α4-1BB in combination induced the highest expression of NKG2D and granzyme B in CD8 T cells. NKG2D, granzyme B and perforin upregulation was tumor independent because the same pattern was observed in all treatments without tumor inoculation (Fig. 3b, c). Correspondingly, IPA indicates that gene sets of cytotoxic T cell development are significantly upregulated in SV plus α4-1BB mAb. These genes include Gzmb (granzyme B), Prf1 (perforin) and Klrk1 (NKG2D) (Fig. 3d). These data indicate that SV plus α4-1BB mAb markedly enhanced cytotoxic T cell activity.

### SV plus α4-1BB mAb induced IFNγ production from T cells

**Fig. 4:**
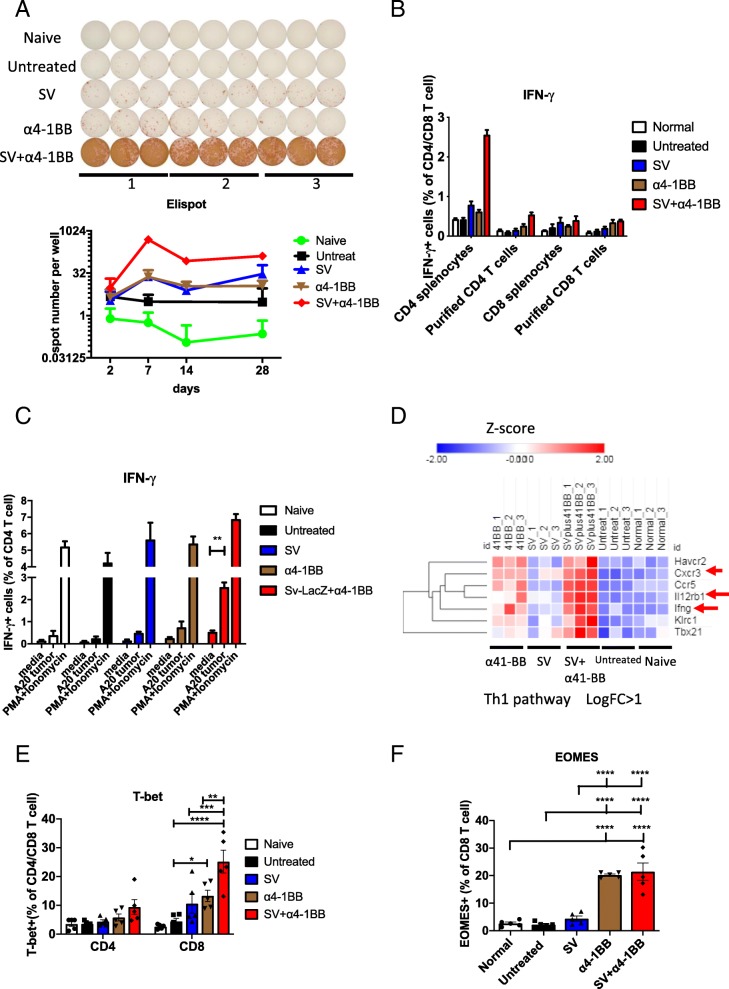
Sindbis virus plus α4-1BB combination induced Th1 differentiation and IFNγ production. **a** IFNγ Elispot analysis of splenocytes harvested at day 2, 7, 14 and 28 from all groups as indicated. Upper panel, IFNγ Elispot image on day 7 after treatment. 1,2,3: three individual mice. Lower panel, IFNγ spots number from indicated groups over the course of treatment (2 × 10^5^ splenocytes per well). No stimulator was added. **b** IFNγ production from CD4/CD8 T cell population in splenocytes and purified CD4/CD8 T cells. All groups were cultured in media for 5 h in the presence of brefeldin A. **c** IFNγ production from purified CD4 T cells at different stimulation conditions. **d** Upregulated Th1 pathway gene set under SV, α4-1BB and SV + α4-1BB stimulation. Expression values are shown by Z-score. Genes are hierarchically clustered by one minus Pearson correlation (day 7). **e** T-bet expression for all groups as indicated. **f**, EOMES expression for all groups as indicated. CD8 T cell gated. **e**, **f** day 7 after treatment. *, *p* < 0.05; **, *p* < 0.01, ****, *p* < 0.0001

Other upregulated genes in the SV plus α4-1BB mAb combined treatment include STAT4 (Fig. 3d) and IL12rb1 (Fig. 4d), which are required for the development of Th1 cells from naive CD4+ T cells and IFNγ production (Fig. 3d) in response to IL-12 [26]. Consistent with this observation, splenocytes from SV plus α4-1BB mAb treatment produced significantly higher number of IFNγ spots compared with other groups, reaching peak production on day 7 (Fig. 4a, upper panel). After day 7, the response dampened but still remained at the highest level compared with other groups (Fig. 4a, lower panel). This is in line with increased IFNγ RNA levels. To identify if TAA or viral antigen induces IFNγ production on day 7, the same experiment was performed in mice not inoculated with tumor cells. For both SV or SV plus α4-1BB treatment, the presence or absence of tumor did not significantly affect IFNγ levels (Additional file 1: Figure S5), confirmeing that IFNγ production on day 7 was mainly an anti-viral response. To identify whether T cells or antigen presentation cells (APCs) play the major role in IFNγ production, we harvested SV treated splenic T cells and naive T cells respectively. T cells from SV treated mice were co-cultured with naive APCs. Conversely, APCs from SV treated mice were cultured with naive T cells. T cells from SV treated mice produced IFNγ when co-cultured with naive APC. Naive T cells produce much less IFNγ spots when cultured with SV infected APC. However, neither T cell nor APC alone could produce elevated numbers of IFNγ spots. These observations indicate that T cells play the dominant role in IFNγ production during SV infection (Additional file 1: Figure S6A). APCs are essential for helping T cells to produce IFNγ.

Next, to identify whether CD4 or CD8 T cells produce IFNγ, flow cytometric analysis was performed for cytokine analysis. Among splenocytes, 2–2.5% SV plus α4-1BB mAb treated CD4 T cells produced IFNγ, which is significantly higher than other groups. Very low percentages of CD8 T cells produced IFNγ in all groups (Fig. 4b). There were much less IFNγ producing T cells after removing APC (Fig. 4b). Also, there was no difference among all groups for IFNγ production. This suggests that T cell-APC interaction is essential for IFNγ production.

To test the antitumor IFNγ production activity of the purified T cells, they were co-cultured for 5 h with A20 cells, which express major histocompatibility complex (MHC) I and II molecules [27]. Only CD4 T cells from the SV plus α4-1BB mAb group produced IFNγ after co-culture (Fig. 4c, Additional file 1: Figure S6B). This indicates that SV plus α4-1BB mAb induces anti-tumor IFNγ production activity. Besides IFNγ, several Th1 associated genes were also upregulated in the T cells from SV plus α4-1BB mAb treated groups. These include Ccr5, Cxcr3, Havcr2(Tim3), IL12rb1 and Klrc1 (Fig. 4d).

T-bet is the key transcription factor which is essential for type 1 immune response (IFNγ production, T cell cytotoxicity) and memory T cell differentiation. In correspondence with the IFNγ expression findings, we observed that SV plus α4-1BB mAb coordinately upregulates T-bet in T cells on day 7 (Fig. 4e). This suggests that SV helps α4-1BB boost the type 1 immune response, which is critical for controlling tumor growth. SV or α4-1BB mAb alone could not induce high IFNγ production due to low T-bet upregulation. Eomesodermin (EOMES), another important transcription factor, is upregulated in activated T cells and is essential for memory CD8 T cell development. Both α4-1BB mAb and SV plus α4-1BB mAb induced high expression of EOMES on day 7 (Fig. 4f). The lack of both T-bet and EOMES results in a lower expression of CXCR3 in T cells and a drastic decrease in the number of tumor-infiltrating T cells [28]. Our data are consistent with these observations. We find elevated CXCR3 (Fig. 4d), T-bet and EOMES (Fig. 4e and f) in T cells of the combined SV plus α4-1BB mAb treated animals.

### SV and α4-1BB mAb stimulated chemotaxis, adhesion and enhanced T cell infiltration and activation in tumor

**Fig. 5:**
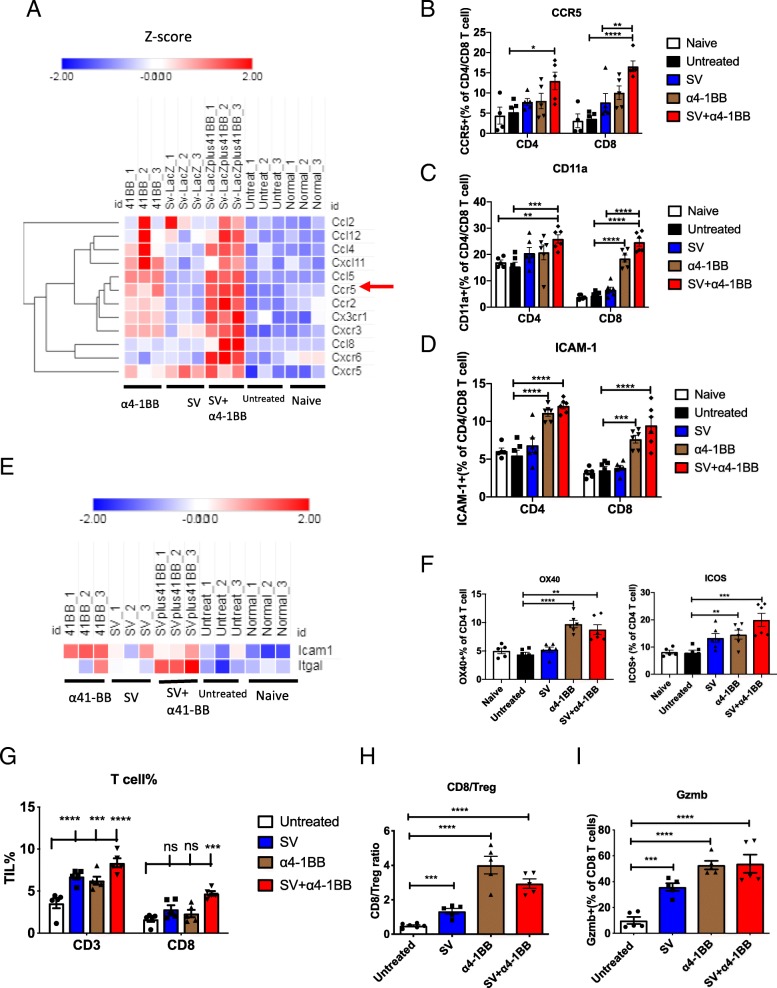
SV and α4-1BB mAb stimulated chemotaxis, adhesion and enhanced T cell infirtration and activation in tumor. **a** Heat map of the expression pattern of SV + α4-1BB upregulated chemokine and chemokine receptor genes (Expression values are shown by Z-score.) Genes are hierarchically clustered by one minus Pearson correlation (day 7). **b** the percentage of CCR5^+^ cells was measured by flow cytometry (day 7). **c**, **d** The percentage of CD11a^+^ (**c**) and ICAM-1^+^ cells (**d**) was measured by flow cytometry. **e** the relative expression of CD11a (ltgal) and ICAM-1 was shown by heat map measured by RNA-Seq. Expression values are shown by Z score. **f** The percentage of OX40^+^ and ICOS^+^ T cells were measured by flow cytometry. *, *p* < 0.05; **, *p* < 0.01; ***, *p* < 0.001; ****, *p* < 0.0001. **g** The frequency of CD3 and CD8 T cells to total harvested cells from tumor was measured by flow cytometry. **h** CD8/Treg ratio of tumor infirtrated T cells. **i** The percentage of granzyme B+ CD8 T cells as indicated. *, *p* < 0.05; **, *p* < 0.01; ***, *p* < 0.001; ****, *p* < 0.0001

Through RNA-Seq, a series of chemokines and chemokine receptors have been identified to be upregulated in SV plus α4-1BB mAb (Fig. 5a). Among those molecules, CCR5 upregulation was confirmed by flow cytometry (Fig. 5b). CCR5 potentiates CD4 T helper cell functions boosting overall anti-tumor responses [29]. We found SV plus α4-1BB significantly upregulates CD11a and ICAM-1(CD54). These two adhesion molecules are highly expressed on activated T cells. LFA-1 (CD11a/CD18)-ICAM-1 interaction is essential for the formation of immune synapses between T cell and APC [30]. LFA-1 and ICAM-1 are also required for T cell-T cell homotypic aggregation and activation [31, 32]. α4-1BB mAb stimulation induced significant upregulation of CD11a and ICAM-1 in both CD4 and CD8 T cells whereas SV does not (Fig. 5c-e). In addition, T cell costimulatory molecule, OX40, was also significantly upregulated in T cells of mice treated with α4-1BB. (Fig. 5f, left). OX40 engagement promotes effector T cell function and survival [33]. ICOS, another CD4 T cell costimulatory molecule, was upregulated in SV or α4-1BB alone but upregulated most in the SV plus α4-1BB combination treatment, suggesting a synergistic effect exists (Fig. 5f, right).

TIL play a critical anti-tumor role and is an important marker for prognosis. Compared with untreated, the percentage of CD3 and CD8 T cells were increased about 2 fold after combination treatment (Fig. 5g). Ki67 were upregulated in those T cells which indicated active division (Additional file 1: Figure S7A). For untreated TIL, the frequency of Foxp3+ Treg cells was the highest (Additional file 1: Figure S7B) and CD8/Treg ratio was the lowest (Fig. 5h). Treatment enhanced the T-bet and EOMES expression in T cells (Additional file 1: Figure S7C,D). NKG2D and granzyme B were highly upregulated in tumor infirtrating CD8 T cells (Fig. 5i, Additional file 1: Figure S7E). Overall, these data indicate that combination treatment enhanced T cell infiltration, division, activation, cytotoxicity and downregulated the inhibitory Treg population.

### SV and α4-1BB mAb synergistically enhanced oxidative phosphorylation

**Fig. 6:**
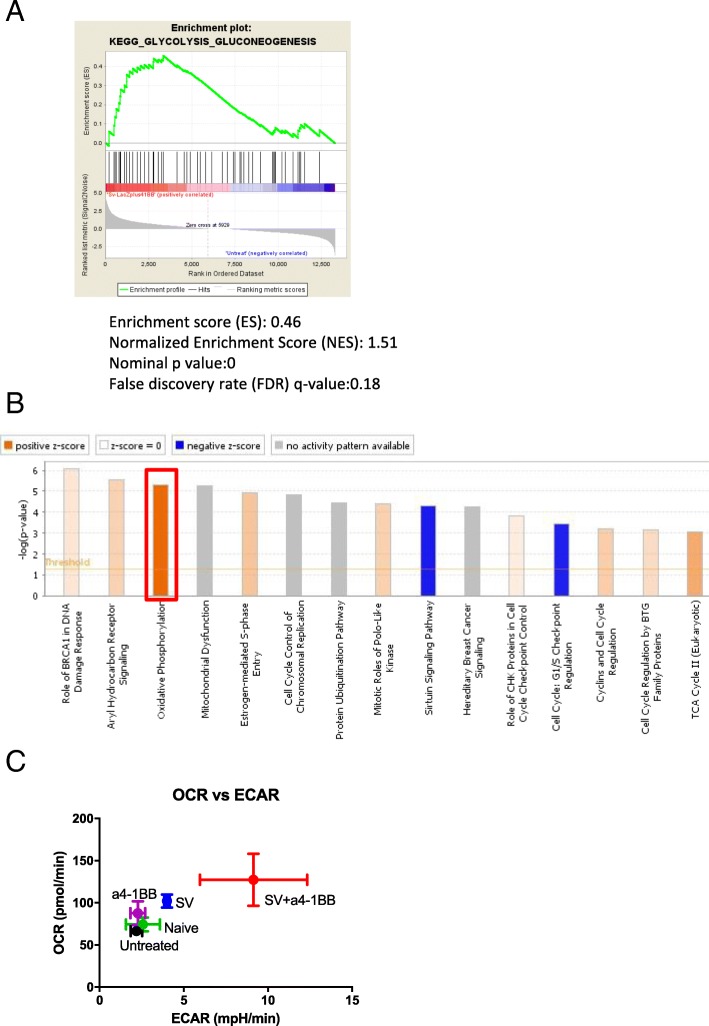
Sindbis virus plus α4-1BB synergistically enhanced T cell glycolysis and oxidative phosphorylation. **a** GSEA enrichment plot of KEGG (SV + α4-1BB vs. untreated) glycolysis pathway. **b** The canonical pathways of SV plus α4-1BB Ab stimulation are clustered by IPA. Red square, oxidative phosphorylation. **c** Both oxygen consumption rate (oxidative phosphorylation) and Extracellular Acidification Rate (glycolysis) were measured by seahorse XFe24. All groups are as indicated (*n* = 4)

T cell activation requires a quick consumption of energy through both enhanced glycolysis and oxidative phosphorylation [34]. Metabolic switch is a major feature of T cell activation and memory T cell development [35]. GSEA KEGG analysis identified that the glycolysis gene set is upregulated in SV plus α4-1BB vs. untreated samples (Fig. 6a). This process quickly produces ATP and supports T cell migration and cytotoxicity in hypoxic or acidific microenvironments. IPA confirms that SV plus α4-1BB mAb synergistically enhanced oxidative phosphorylation (Fig. 6b).

We assessed both oxygen consumption rate (OCR, represents oxidative phosphorylation) and extracellular acidification rate (ECAR, represents glycolysis) of all groups (Fig. 6c). Compared with other groups, SV plus α4-1BB significantly increased both OCR and ECAR. This indicates that both glycolysis and oxidative phosphorylation are activated in T cells of animals treated with SV plus α4-1BB.

### SV plus low dose α4-1BB mAb cured A20 tumor bearing mice

To reduce the potential risk of cytotoxicity and expense of treatment with SV vectors plus α4-1BB, we explored whether low doses of α4-1BB mAb and fewer injections would be as effective in curing tumor bearing mice as the higher doses and frequencies used in our initial stduies. As demonstrated (Additional file 1: Figure S8A and B), A20 tumor bearing mice can be completely cured by SV (3 times per week for 3 weeks) plus a low dose of α4-1BB mAb (50μg per week for 3 weeks). This reduces both the SV and α4-1BB mAb dosing requirements. The reduced dose of α4-1BB mAb would be helpful, as well, in preventing the α4-1BB mAb induced liver toxicity reported by some investigators [36].

### All tumor cured mice acquired long lasting antitumor immunity

**Fig. 7:**
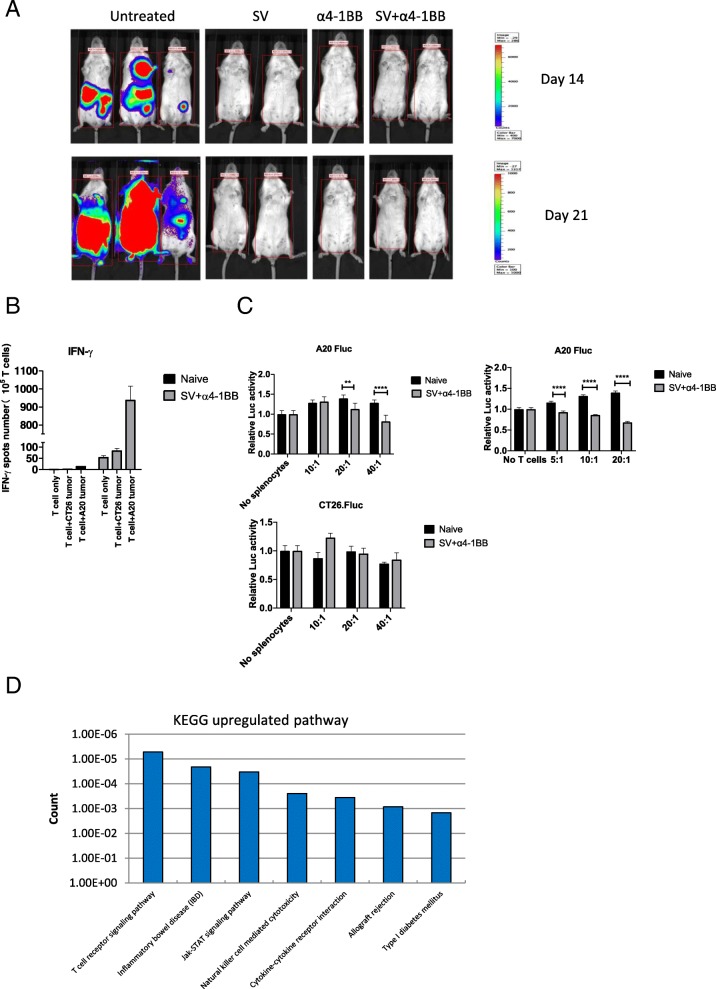
Cured mice are completely protected from A20 lymphoma rechallenge. **a**, Bioluminescence images of groups, previously treated as indicated, were rechallenged with A20 lymphoma cells. **b** IFNγ production from purified T cells of all groups(To SV + α4-1BB, 4 months after treatment finished), in the absence or presence of A20 tumor cells (5 × 10^4^ per well), was measured by Elispot assay. **c** Cytotoxicity assay was performed the same as Fig. 3a. Left 2 panels, total splenocytes were used. Right, purified T cells were used. Left upper, A20 Fluc cells and left lower, CT26 Fluc cells were used for co-culture. **d**, Significant differential (SD) upregulated gene sets are clustered by DAVID KEGG analysis. *, *p* < 0.05; **, *p* < 0.01; ****, *p* < 0.0001

To investigate the memory response to A20 lymphoma, naïve and tumor cured mice were inoculated with 3 × 10^6^ A20 tumor cells. Only mice that had survived more than 4 months after 1st time of tumor challenge were chosen. In all tumor cured mice, we found that A20 lymphoma was completely rejected whereas naïve mice were susceptible to A20 inoculation (Fig. 7a).

To confirm anti-tumor specificity has been elicited, we measured IFNγ production of purified T cells in the presence or absence of tumor cells by Elispot assay. T cells were isolated from naïve and cured mice under SV plus α4-1BB treatment (4 months after treatment finished). Isolated T cells were co-cultured with A20 and CT26 tumor cells respectively. Co-culturing with A20 cells dramatically enhanced IFNγ production, whereas co-culturing with CT26 cells only slightly enhanced IFNγ production (Fig. 7b).

Next, we measured cytotoxicity to both naïve and cured mice under SV plus α4-1BB treatment (the same method as Fig. 3a). Compared with naïve, cured mice had enhanced cytotoxicity to A20 lymphoma cells, but not to CT26 tumor cells. To confirm that this is mediated by T cells, the same experiment was done using purified T cells. Cured mice had enhanced cytotoxicity compared with naïve mice (Fig. 7c).

To better understand differences between this memory T cell response and the initial treatment responses as observed on day 7, RNA-Seq was performed by using purified splenic T cells from all rechallanged groups. In T cells of these rechallened mice we found only a few differentially expressed genes among the three treated groups (Additional file 6: Table S5), indicating that tumor cured mice develop a very similar T cell gene expression profile regardless of treatment method. Compared with untreated, KEGG analysis indicates that TCR signaling is the highest upregulated pathway in SV plus α4-1BB group (Fig. 7d), indicating that continuously enhanced TCR signaling is critical for maintaining antitumor immunity.

## Discussion

The conventional view of oncolytic virus therapy against tumors is that it requires selective infection of cancer cells resulting in the induction of cancer cell lysis and apoptosis. TAAs, released from dead tumor cells, attract and further stimulate an antitumor immune response. Although A20 lymphoma cells are resistant to infection by SV, these vectors offer an unique opportunity to treat unsusceptiable liquid tumors efficiently. This inducing long-lasting memory/ anti-immunity regardless of infectivity. In previous studies, we demonstrated vectors encoding TAAs, such as NYESO1, could cure CT26-NYESO1 tumors [6, 18]. Here, we found that encoding a TAA is not necessary for SV vectors plus α4-1BB mAb therapy to be fully successful. SV vectors lacking an A20 lymphoma TAA were able to treat A20 lymphoma and, in combination with α4-1BB mAb, eradicated the growing tumors.

Compared with other cancer immunotherapies, SV therapy has several prominent advantages. Unlike conventional CAR-T, TCR-T or neoantigen specific patient derived T cells, SV therapy does not require long processing time including cell harvesting, expansion, gene editing and reinfusion. SV therapy also eliminates concerns of graft versus host disease involved in using allogenic T cells.

SV therapy does not require the incorporation of specific TAAs as does tumor antigen vaccine. This is particularly important when effective immune reactive TAAs are unknown. It is possible that the immunotherapeutic response of SV vectors plus α4-1BB mAb is independent of whether a tumor is “cold” (i.e., having few TAAs or mutation-specific neoantigens capable of promoting robust T cell activation) or “hot.”

Clinical trials of 2 4-1BB agonist antibodies, urelumab and utomilumab, are underway. Despite initial signs of efficacy, clinical development of urelumab has been hampered by inflammatory liver toxicity at doses > 1 mg/kg [37]. Utomilumab has a superior safety profile, but is a less potent 4-1BB agonist relative to urelumab [37]. Both antibodies have demonstrated promising results in patients with lymphoma and are being tested in combination therapy trials with other immunomodulatory agents [37]. The combination of α4-1BB mAb with other immunomodulatory reagents like SV vectors might help overcome these limitations and should be explored.

The quick inhibition of tumor growth is critical for cancer therapy because tumor cells undergo exponentially rapid division. However, the induction of adaptive immunity and establishment of tumor specific immunity takes a long time. An ideal therapy requires an early, quick reduction of tumor burden, and a later induction of anti-tumor specificity that prevents relapse. In this study, we proposed that SV plus α4-1BB mAb treatment induced massive T cell activation due to viral induced immune response. This massive activation helps to control the tumor in a TAA nonspecific manner. A similar mechanism has been demonstrated in several other studies. Morphy et al. showed that combining agonistic anti-CD40 with IL-2 induces expansion of highly cytolytic, antigen-independent “bystander-activation” that was responsible for anti-tumor effects [38, 39].

In another study, cytokine was shown to directly induce memory CD8 T cells expressing NKG2D and granzyme B and that these T cells acquire broadly lytic capabilities without cognate antigen engagement [40]. In our system, we found that both NKG2D (KLRK1) and granzyme B are highly expressed under combination treatment. This massive nonspecific activation is critical for controlling tumor growth at an early time point (day 7). This step is also important for inducing anti-tumor specificity that is mediated by TAAs released from dead tumor cells due to nonspecific killing. After tumor regression, T cells from treated animals were able maintain the ability to produce IFNγ and acquired immunological memory to rapidly reject A20 lymphoma rechallenges. IFNγ production from purified T cells of cured mice was significantly enhanced after encountering A20 tumor cells. This demonstrates that anti-tumor specificity is fully established in cured mice.

Oncolytic vaccinia virus and adenovirus have been used in combination with 4-1BB agonist (either α4-1BB mAb or OV expressing 4-1BBL) by other investigators. John et al. [41] demonstrated that vaccinia virus and anti-4-1BB combination therapy elicits strong antitumor immunity. However, this combination therapy did not cure mice. While, vaccina virus can infect tumor cells and induce lytic cell death, in our model, SV infection of tumor cells is not necessary. In another vaccinia virus therapy, oncolytic vaccinia virus expressing 4-1BBL has been used for treatment [42]. However the effects were only observed when combined with host lymphodepletion [42]. While lymphodepletion is commonly used in some immunotherapies, it can lead to toxicity, and increased risks of infection. Adenovirus has also been used with α 4-1BB agonist in a combination therapy. Huang et al. [43] showed that tumor specific immunity was induced by the combination of dendritic cells (DC) and oncolytic adenovirus expressing IL-12 and 4-1BBL. DC co-transfer is required for this therapy. The extra cost and time consumption involved in self-DC harvesting, culture and maturation might pose barriers to application of this approach in cancer patients, whereas, our combination therapy does not require any ex-vivo steps. In another study, oncolytic adenovirus armed with CD40L and 4-1BBL were used to treat pancreatic cancer. Again, OV infection of tumor cells and tumor associated stromal cells was a prerequisite for antitumor effect and immune activation [44], a condition that is not required by SV vectors.

Our study identified and compared the upregulated molecular pathways of responsive T cells induced by SV vectors and α 4-1BB mAbs alone and in combination. These observations provide novel insights to guide future studies.

In summary, OV therapy has become a promising therapy for treating cancer. The combination of oncolytic virus and checkpoint inhibitor generally achieve a better therapeutic effect than either alone [18, 45]. α4-1BB mAb has also been used in combination with other therapeutic agents to enhance its antitumor effect [46, 47]. In this study, we demonstrated that the combination of SV and α4-1BB mAb has a synergistic effect and represents a potent and robust therapeutic treatment able to cure B lymphomas and provide long term protection in a preclinical model.

## Conclusions

In conclusion, SV vectors in combination with α4-1BB mAb completely eradicated a B-cell lymphoma in a preclinical mouse model, a result that could not be achieved with either treatment alone. Tumor elimination involves a synergistic effect of the combination that significantly boosts T cell cytotoxicity, IFN-γ production, migration, tumor infiltration and oxidative phosphorylation. In addition, all mice that survived after treatment developed long lasting antitumor immunity. Our study provides a novel, alternative method for B cell lymphoma treatment and describes a rationale to help translate SV vectors plus agonistic mAbs into clinical applications.

## Additional files

Additional file 1: **Figure S1.** A20 lymphoma cells were SV infection resistant. A, A20 cells and BHK cells were infected with SV carrying GFP overnight. GFP expression was observed under fluorescent microscope. B, SV-GFP infectivity to BHK cells was verified by flow cytometry. C, SV-GFP infectivity to A20 cells in vivo were measured by flow cytometry. 10^7^ A20 cells (express CD45.2) were inoculated to CByJ.SJL(B6)-Ptprca/J (CD45.1 BALB/C) mice. Recipient mice were treated with SV-GFP 4 days later. GFP expression was measured the next day. **Figure S2.** SV infection enhanced cell cycle progression and migration. A, DAVID KEGG analysis. B, GSEA enrichment plot of KEGG (SV vs. Untreated) cell cycle pathway (SV vs. Untreated). C, cell movement pathway was signficiantly enhanced by IPA(SV vs. Untreated). **Figure S3.** Significant differential (SD) upregulated genes are clustered by DAVID analysis. **Figure S4.** Untreated group had low ratio of T cells and high ratio of regulatory T cells on day 28. The frequency of CD4 (A), CD8 (B), Treg (C) were measured by flow cytometry. D, Treg/CD8 ratio as indicated. **Figure S5.** IFNγ production from splenocytes of all groups with or without tumor inoculation on day 7 after treatment was measured by Elispot. With tumor: tumor was inoculated on day 0. Without tumor: tumor was not inoculated. No stimulator was added in Elispot assay. **Figure S6.** IFNγ production measurement. A, IFN**γ** production (at day 7) by all groups, as indicated, was measured by Elispot. B, IFN**γ** production of purified T cells (CD8 T cell portion) on day 7 after treatment was measured by flow cytometry. **Figure S7.** The phenotype of tumor infirtrated T cells. A-E, The percentage of Ki67+, Foxp3+, T-bet+, EOMES+, NKG2D+ T cells were measured by flow cytometry. **Figure S8.** SV plus low dose α4-1BB mAb cured A20 tumor bearing mice. (PPTX 9838 kb)

Additional file 2: **Table S1.** The SD expressed genes list for SV vs. untreated group by RNA-Seq (q < 0.05, Log2FC ≥ 1 and Log2FC ≤ − 1). (XLSX 24 kb)

Additional file 3: **Table S2.** The upregulated cell movement pathway for SV vs. untreated group by IPA. SV induced SD upregulated gene sets are clustered by DAVID analysis (SV vs. Untreated). Gene clusters are ranked by enrichment score. (XLSX 9 kb)

Additional file 4: **Table S3.** The SD expressed genes list for SV + α4-1BB vs. untreated group by RNA-Seq (q < 0.05, Log2FC ≥ 1 and Log2FC ≤ − 1). (XLSX 113 kb)

Additional file 5: **Table S4.** The SD expressed genes list for SV + α4-1BB vs. SV group by RNA-Seq (q < 0.05, Log2FC ≥ 1 and Log2FC ≤ − 1). (XLSX 52 kb)

Additional file 6: **Table S5.** The SD expressed gene lists among all tumor cured mice groups. (XLSX 10 kb)

## Data Availability

The datasets used and/or analyzed during the current study are available from the corresponding author on reasonable request.

## Abbreviations

APC: Antigen presentation cell
DAVID: The Database for Annotation, Visualization and Integrated Discovery
ECAR: Extracellular acidification rate
GSEA: Gene Set Enrichment Analysis
IPA: Ingenuity pathway analysis
KEGG: Kyoto Encyclopedia of Genes and Genomes
OCR: Oxygen consumption rate
OV: Oncolytic virus
SD: Significant differential
SV: Sindbis virus
TAA: Tumor associated antigen
TIL: Tumor infiltrating lymphocyte
