# Supplementary material for: Molecular and metabolic pathways mediating curative treatment of a non-Hodgkin B cell lymphoma by Sindbis viral vectors and anti-4-1BB monoclonal antibody
Source: J Immunother Cancer. 2019 Jul 15;7:185. doi: 10.1186/s40425-019-0664-3 (PMC6632218; doi:10.1186/s40425-019-0664-3)
Supplement: Supplementary file 1 — Figure S1. A20 lymphoma cells were SV infection resistant. A, A20 cells and BHK cells were infected with SV carrying GFP overnight. GFP expression was observed under fluorescent microscope. B, SV-GFP infectivity to BHK cells was verified by flow cytometry. C, SV-GFP infectivity to A20 cells in vivo were measured by flow cytometry. 107 A20 cells (express CD45.2) were inoculated to CByJ.SJL(B6)-Ptprca/J (CD45.1 BALB/C) mice. Recipient mice were treated with SV-GFP 4 days later. GFP expression was measured the next day. Figure S2. SV infection enhanced cell cycle progression and migration. A, DAVID KEGG analysis. B, GSEA enrichment plot of KEGG (SV vs. Untreated) cell cycle pathway (SV vs. Untreated). C, cell movement pathway was signficiantly enhanced by IPA(SV vs. Untreated). Figure S3. Significant differential (SD) upregulated genes are clustered by DAVID analysis. Figure S4. Untreated group had low ratio of T cells and high ratio of regulatory T cells on day 28. The frequency of CD4 (A), CD8 (B), Treg (C) were measured by flow cytometry. D, Treg/CD8 ratio as indicated. Figure S5. IFNγ production from splenocytes of all groups with or without tumor inoculation on day 7 after treatment was measured by Elispot. With tumor: tumor was inoculated on day 0. Without tumor: tumor was not inoculated. No stimulator was added in Elispot assay. Figure S6. IFNγ production measurement. A, IFNγ production (at day 7) by all groups, as indicated, was measured by Elispot. B, IFNγ production of purified T cells (CD8 T cell portion) on day 7 after treatment was measured by flow cytometry. Figure S7. The phenotype of tumor infirtrated T cells. A-E, The percentage of Ki67+, Foxp3+, T-bet+, EOMES+, NKG2D+ T cells were measured by flow cytometry. Figure S8. SV plus low dose α4-1BB mAb cured A20 tumor bearing mice. (PPTX 9838 kb) [file 40425_2019_664_MOESM1_ESM.pptx]

## Slide 1
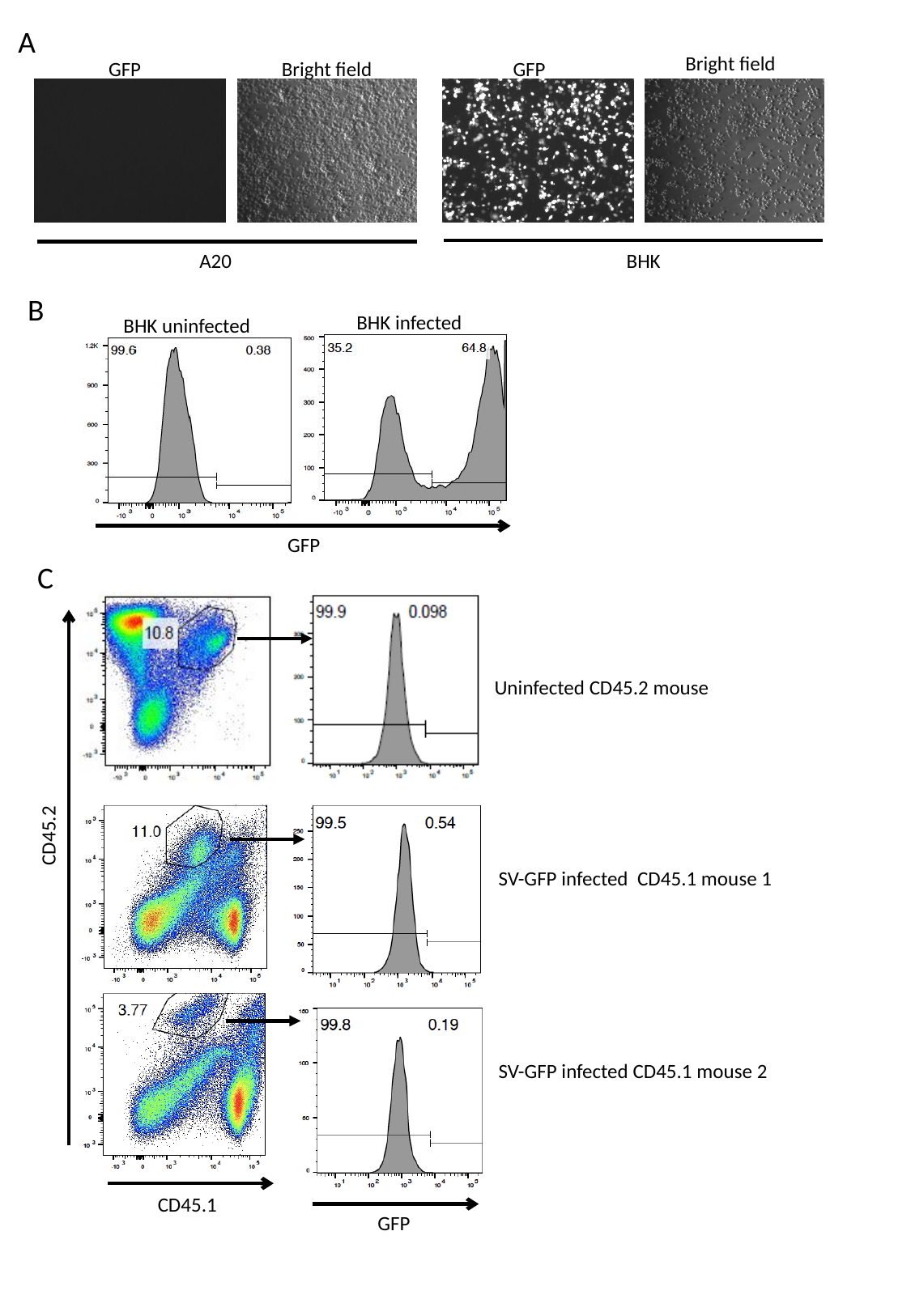

A
Bright field
GFP
Bright field
GFP
A20
BHK
B
BHK infected
BHK uninfected
GFP
C
Uninfected CD45.2 mouse
CD45.2
SV-GFP infected CD45.1 mouse 1
SV-GFP infected CD45.1 mouse 2
CD45.1
GFP

## Slide 2
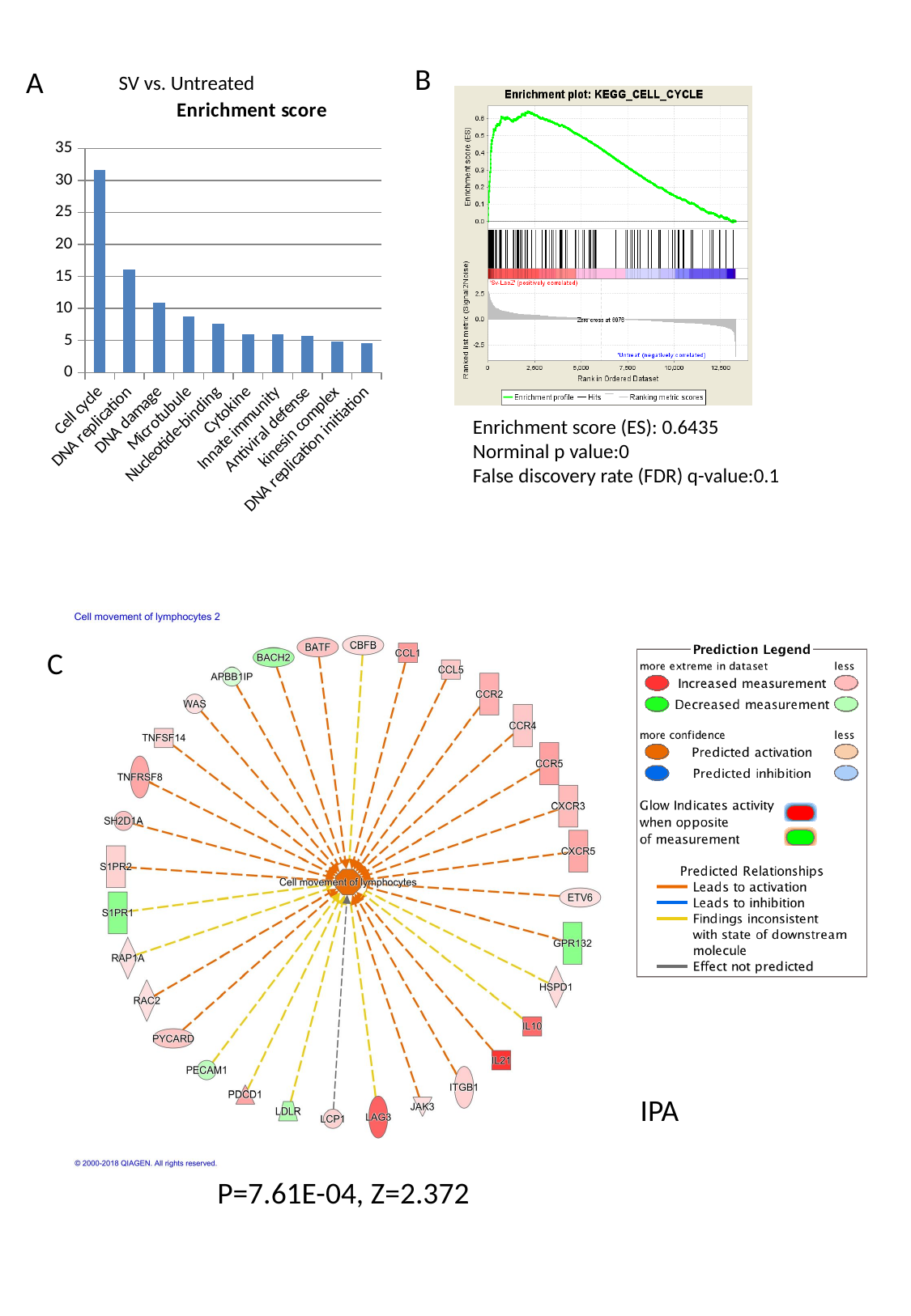

B
Enrichment score (ES): 0.6435
Norminal p value:0
False discovery rate (FDR) q-value:0.1
A
SV vs. Untreated
### Chart:
| Category | Enrichment score |
|---|---|
| Cell cycle | 31.6132561102063 |
| DNA replication | 16.0242589500051 |
| DNA damage | 10.8688580604705 |
| Microtubule | 8.779141209234169 |
| Nucleotide-binding | 7.614920558809 |
| Cytokine | 6.040872007638495 |
| Innate immunity | 6.02167189151969 |
| Antiviral defense | 5.68389940147804 |
| kinesin complex | 4.77546592900992 |
| DNA replication initiation | 4.602285925727505 |
IPA
P=7.61E-04, Z=2.372
C

## Slide 3
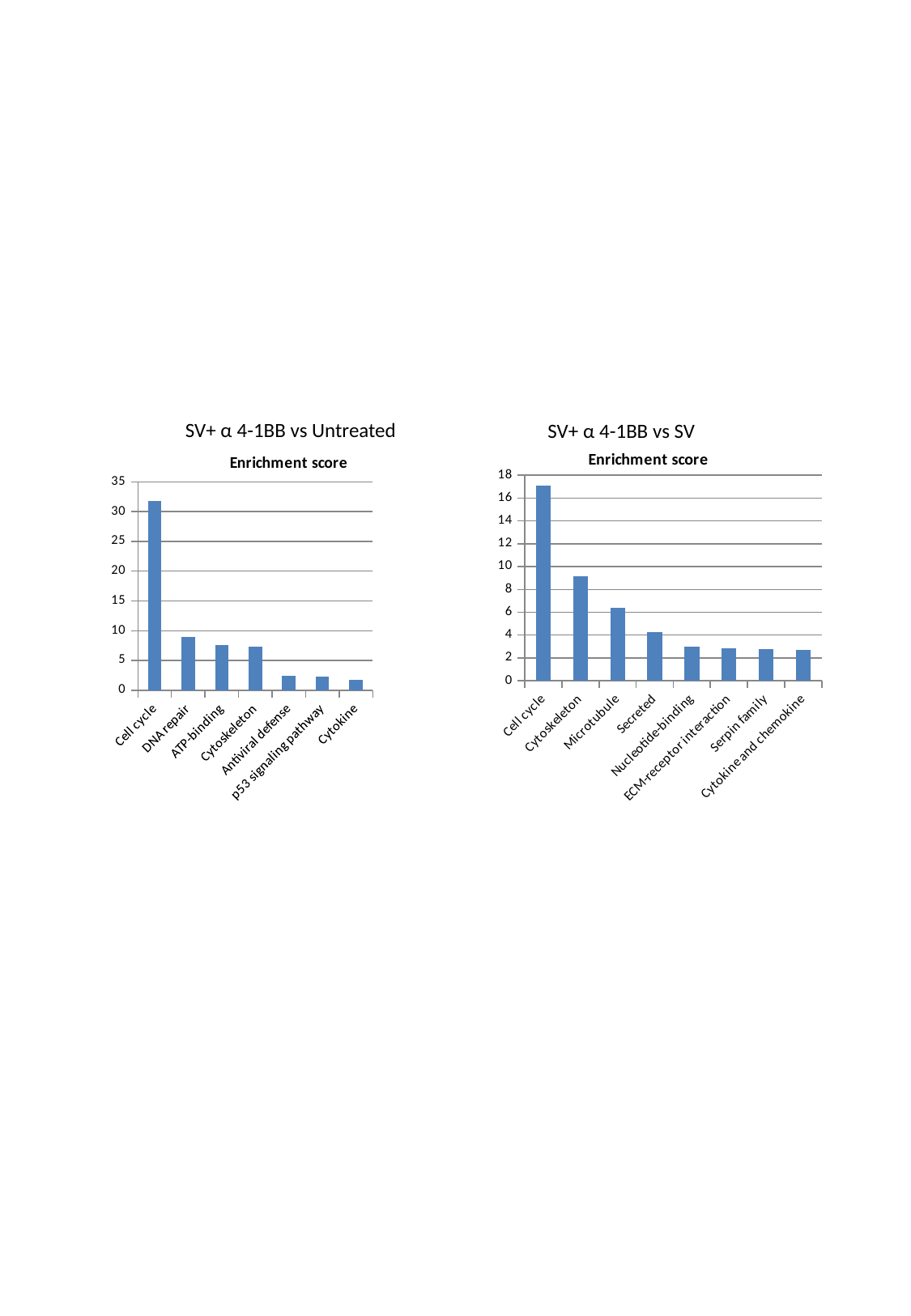

SV+ α 4-1BB vs Untreated
SV+ α 4-1BB vs SV
### Chart:
| Category | Enrichment score |
|---|---|
| Cell cycle | 17.1039009072748 |
| Cytoskeleton | 9.18186458411928 |
| Microtubule | 6.40314123704312 |
| Secreted | 4.23657441344659 |
| Nucleotide-binding | 3.0044364836157 |
| ECM-receptor interaction | 2.837143399123262 |
| Serpin family | 2.75803995645226 |
| Cytokine and chemokine | 2.677096244952 |
### Chart:
| Category | Enrichment score |
|---|---|
| Cell cycle | 31.7598434909465 |
| DNA repair | 8.902578083910818 |
| ATP-binding | 7.648164922755404 |
| Cytoskeleton | 7.29091170497827 |
| Antiviral defense | 2.46214983786037 |
| p53 signaling pathway | 2.36061875874549 |
| Cytokine | 1.79702332283288 |

## Slide 4
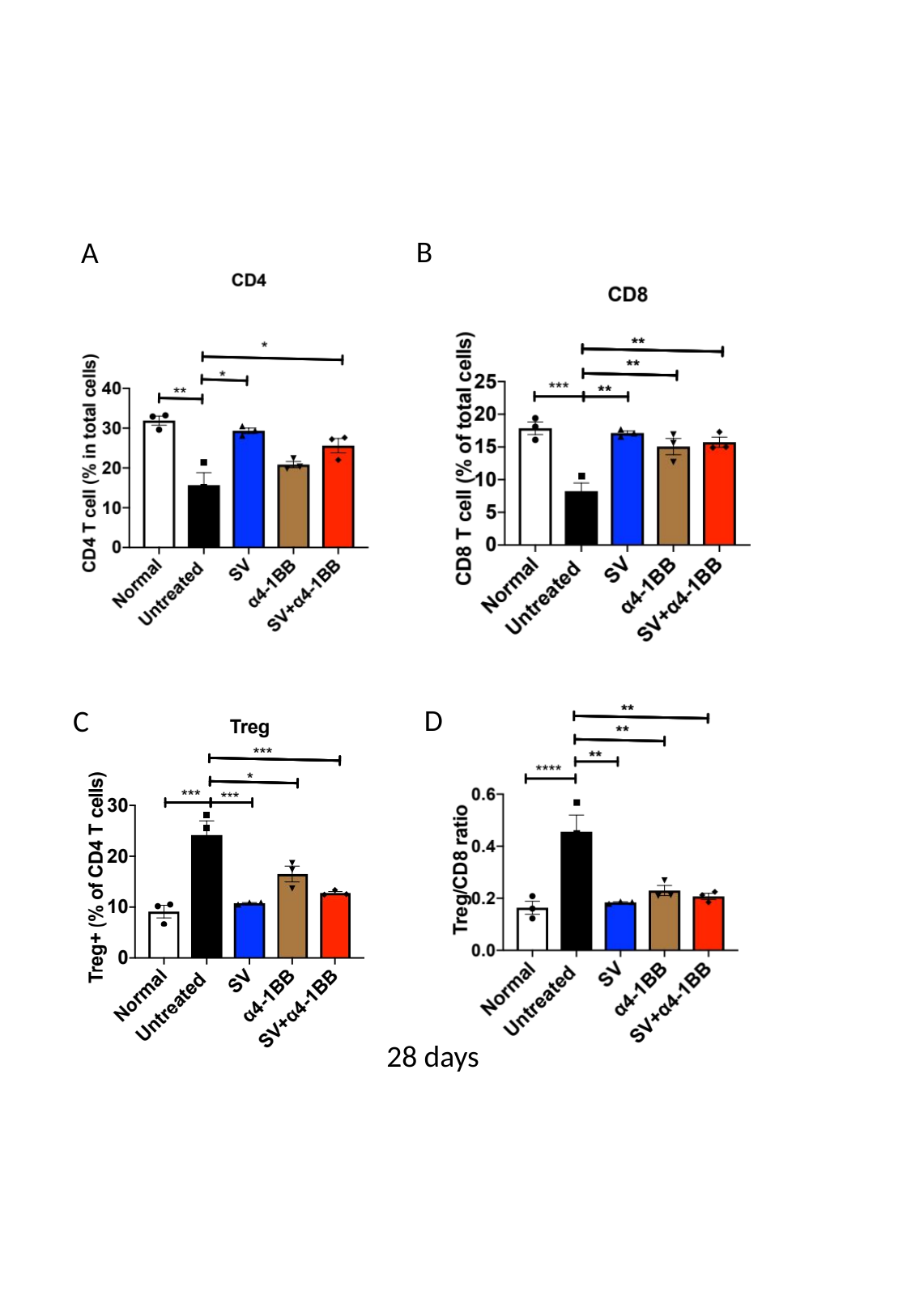

B
A
D
C
28 days

## Slide 5
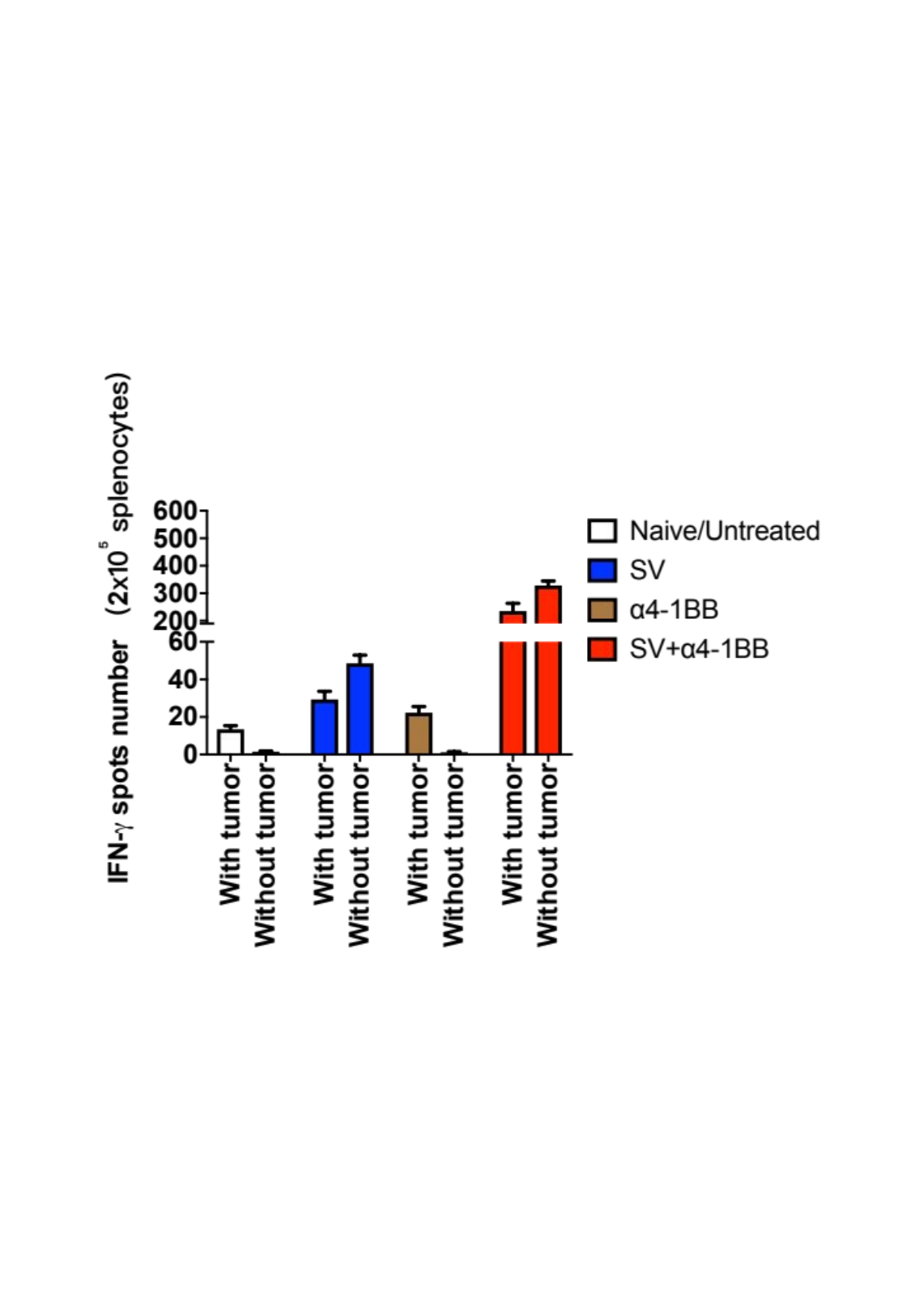

## Slide 6
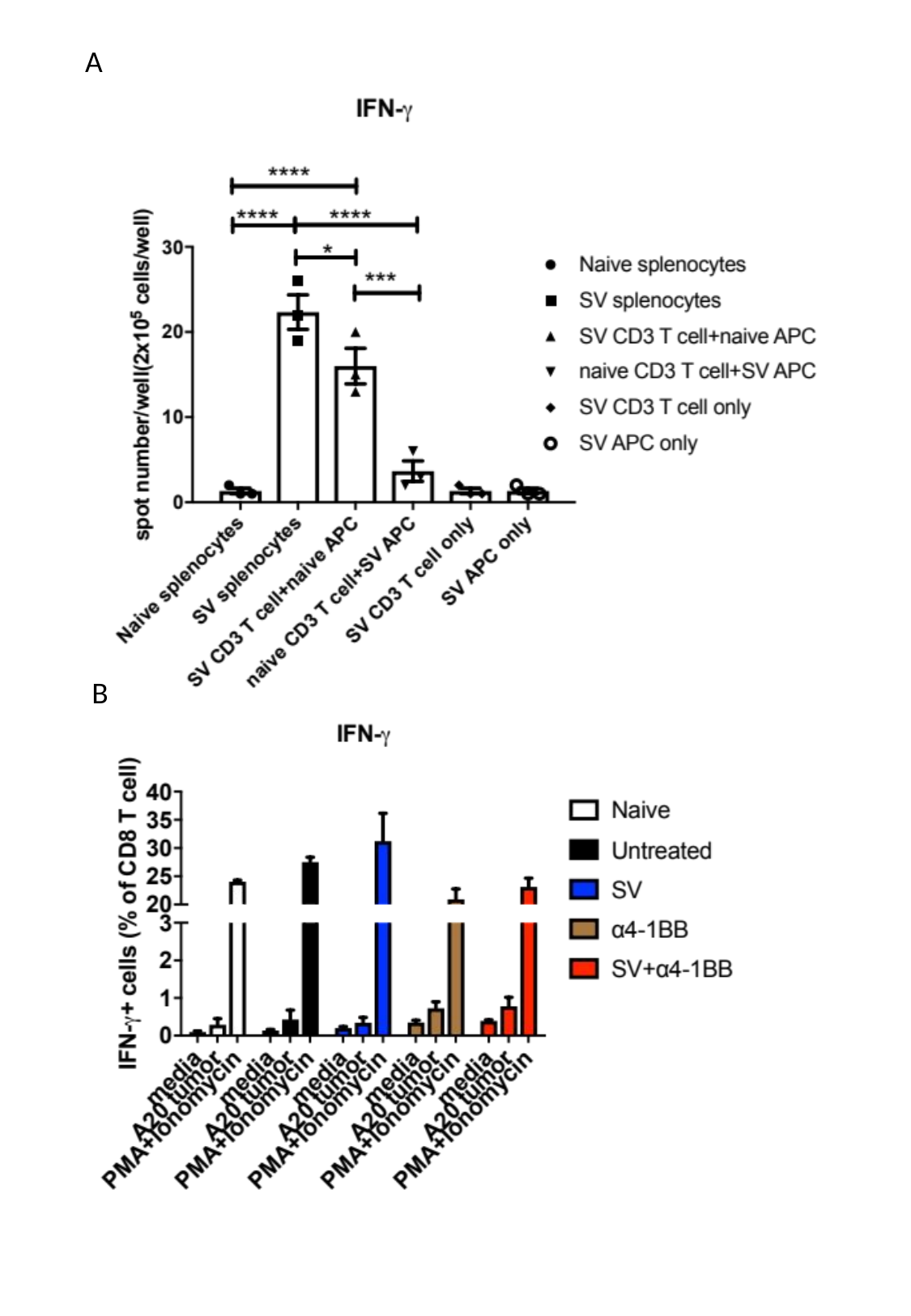

A
B

## Slide 7
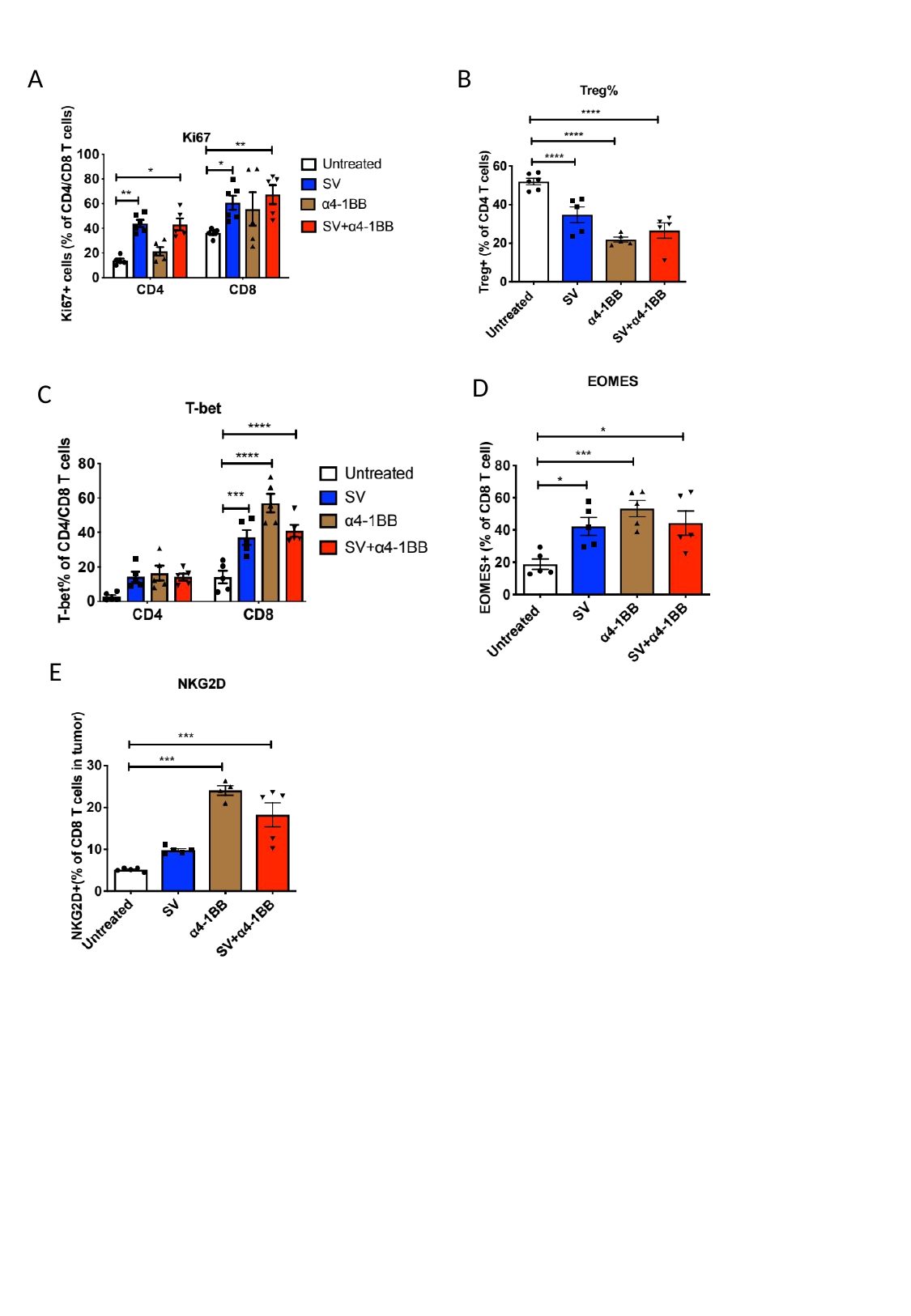

A
B
D
C
E

## Slide 8
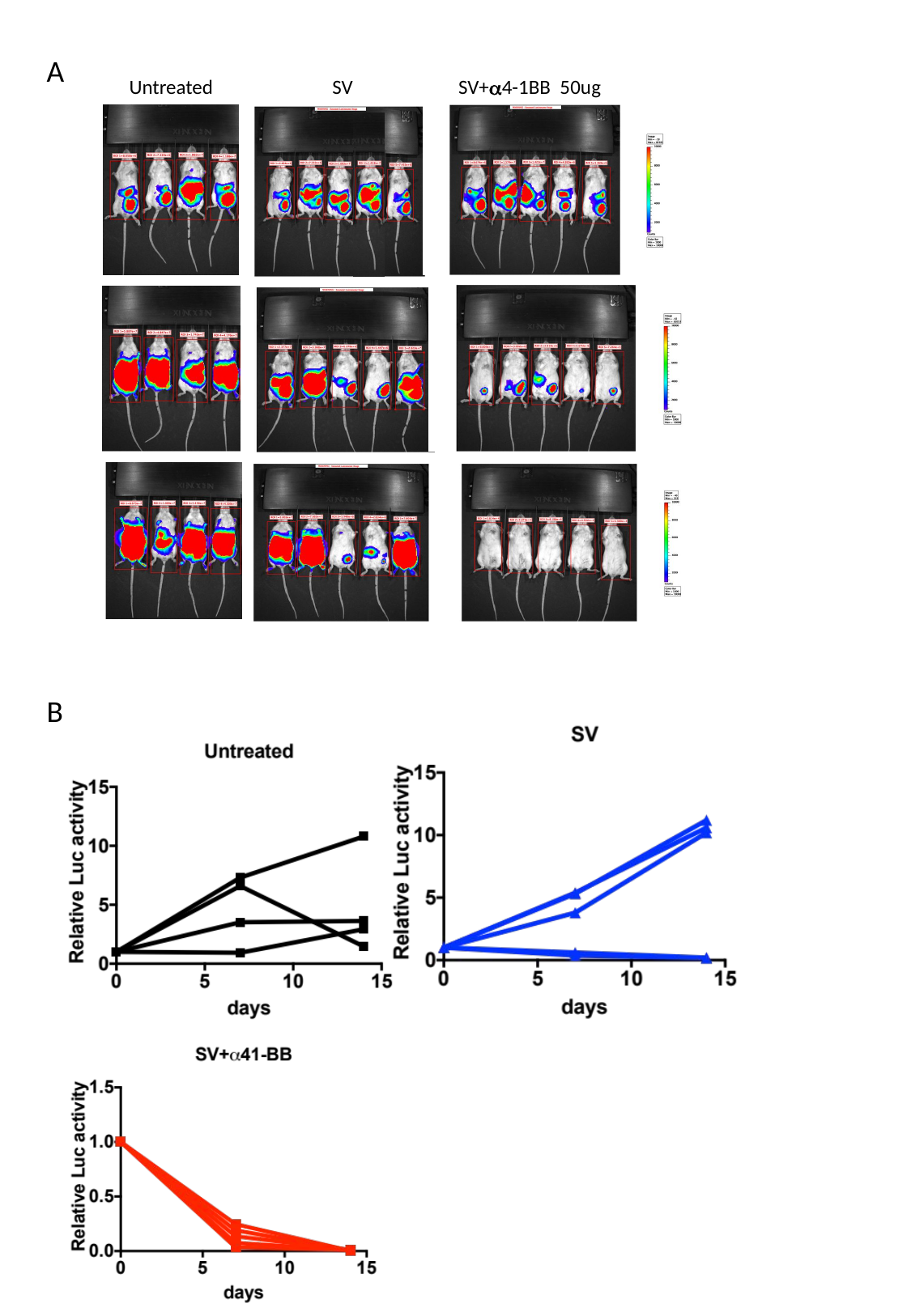

A
SV+α4-1BB 50ug
Untreated
SV
B
